# Supplementary material for: Treatment-induced increase in total body potassium in patients at high risk of ventricular arrhythmias; a randomized POTCAST substudy
Source: PLoS One. 2023 Jul 19;18(7):e0288756. doi: 10.1371/journal.pone.0288756 (PMC10355384; doi:10.1371/journal.pone.0288756)
Supplement: S1 File — (PDF) [file pone.0288756.s001.pdf]

Supplementary appendix

Treatment-induced increase in total body potassium in patients at high risk of ventricular arrhythmias; a randomized POTCAST substudy

S1 Fig: Number of registered gamma rays along the energy spectrum with 40K at 1.46 MeV (red area).

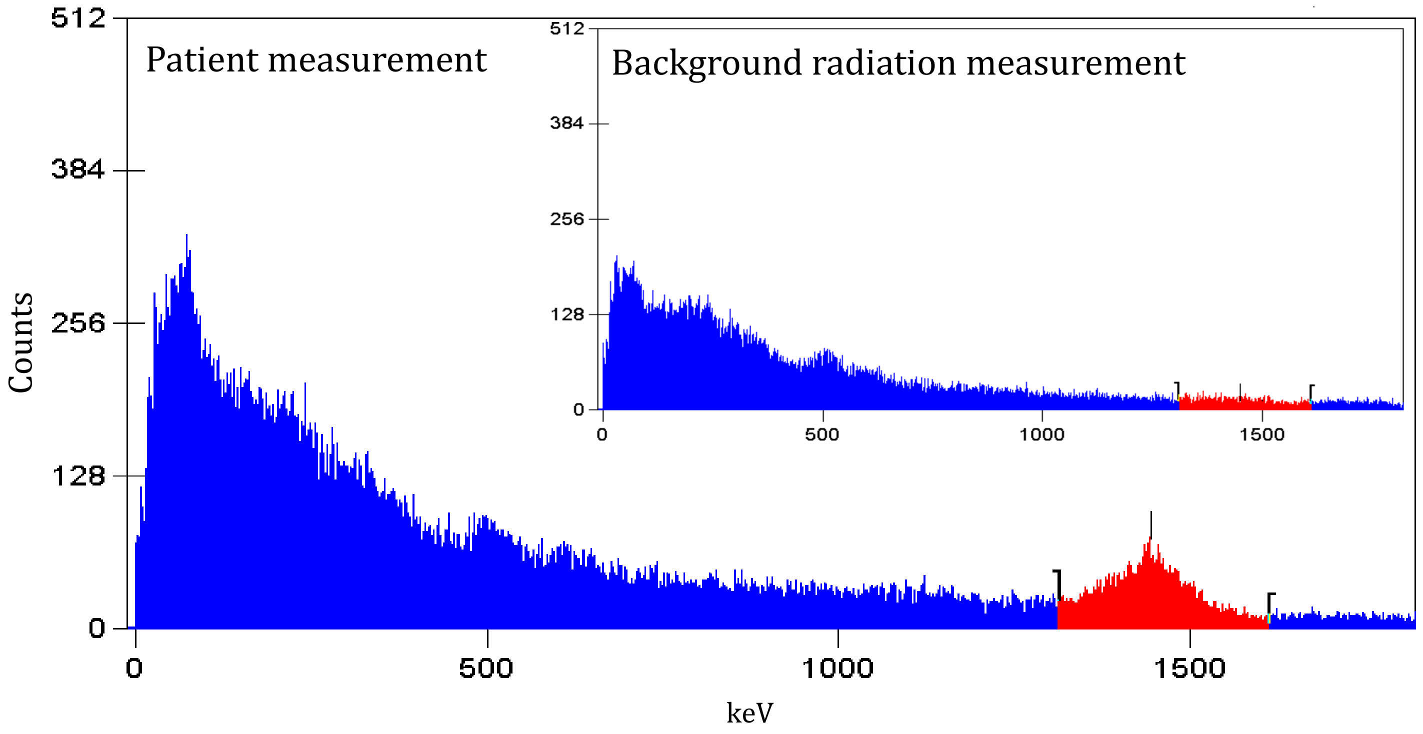

S2 Fig: Bland-Altman plot of the repeated baseline measurement of TBK in all study participants (n=14). Showing mean difference (black line) and 95% limits of agreement (gray lines).

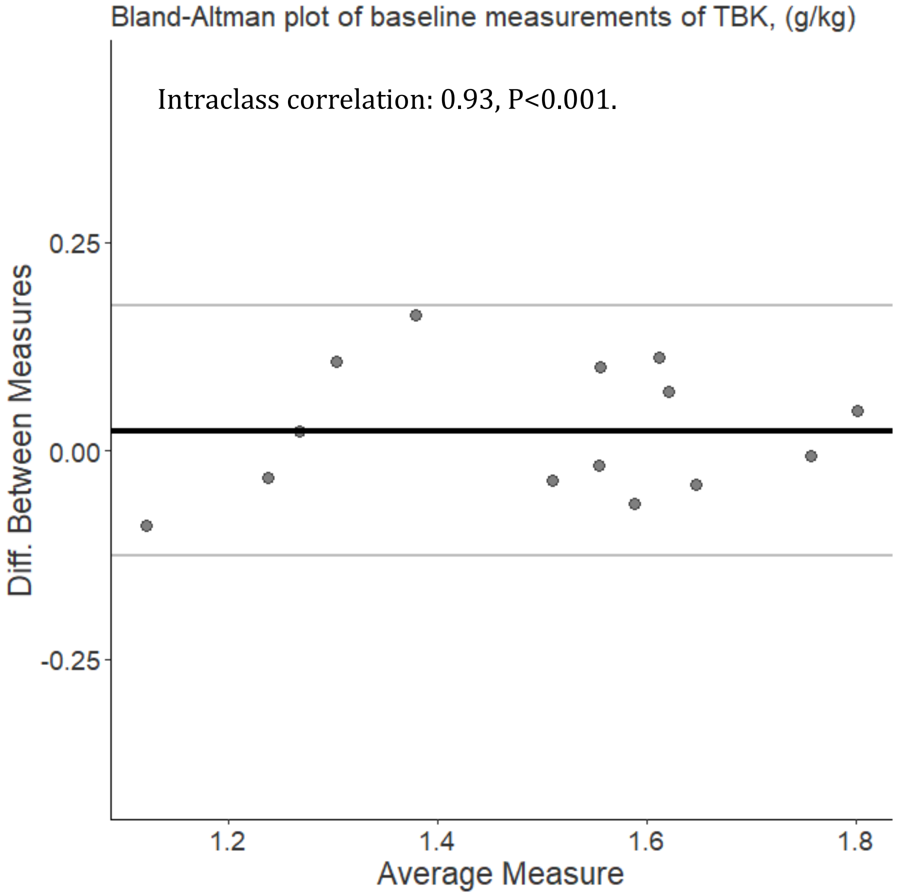

S1 Table: Mean TBK (grams potassium/kg body weight) and p-K (mmol/l) at baseline, six-weeks, and six-months and standard deviations.

Group	Mean TBK, baseline	Mean TBK, six-weeks	Mean TBK, six-months	Mean p-K, baseline	Mean p-K, six-weeks	Mean p-K, six-months
Control	1.56 (±0.17)	1.55 (±0.16)	1.53 (±0.11)	3.8 (±0.2)	3.9 (±0.4)	4.1 (±0.4)
Intervention	1.44 (±0.23)	1.48 (±0.24)	1.45 (±0.16) *	3.8 (±0.1)	4.5 (±0.4)	4.4 (±0.3) *

* One patient missed the six-month measurement due to another illness independent of the trial.
p-K: Plasma Potassium; TBK: Total Body Potassium

S2 Table: p-Na (mmol/l) and p-Mg (mmol/l) at baseline, six-weeks, and six-months.

ID	Group	p-Na, baseline	p-Na, six-weeks	p-Na, six-months	p-Mg, baseline	p-Mg, six-weeks	p-Mg, six-months
1	Control	141	139	140	0.89	0.96	0.94
2	Control	141	141	144	0.88	0.84	0.92
3	Control	141	142	141	0.86	0.91	0.91
4	Control	138	139	141	0.90	1.04	0.98
5	Control	139	143	141	1.0	0.92	0.86
6	Control	144	143	141	0.85	0.89	0.88
7	Control	140	140	142	0.67	0.65	0.74
8	Intervention	145	139	*N/A**	0.90	0.87	*N/A**
9	Intervention	138	139	137	0.79	0.88	0.97
10	Intervention	140	141	141	0.88	0.87	0.89
11	Intervention	140	136	138	0.81	0.84	0.84
12	Intervention	141	139	138	0.91	0.98	0.79
13	Intervention	137	142	139	0.82	0.81	0.82
14	Intervention	139	131	137	0.88	0.80	0.85

* One patient missed the six-month measurement due to another illness independent of the trial.
p-Mg: Plasma Magnesium, p-Na; Plasma Sodium,
