# Supplementary material for: Treatment-induced increase in total body potassium in patients at high risk of ventricular arrhythmias; a randomized POTCAST substudy
Source: PLoS One. 2023 Jul 19;18(7):e0288756. doi: 10.1371/journal.pone.0288756 (PMC10355384; doi:10.1371/journal.pone.0288756)
Supplement: S3 File — (PDF) [file pone.0288756.s003.pdf]

# Raising total body potassium using aldosterone antagonists and oral potassium supplements.

A substudy on "Arrhythmia prevention in high risk cardiovascular patients using targeted potassium levels"-study (the Potassium trial)."

## Background:

### Potassium regulation:

Potassium is a metallic, inorganic ion and is the most abundant cation in the body. Total body potassium (TBK) amounts to approximately 55 mEq/kg body weight of which 98% exists in the intracellular space (primarily in muscle-, skin-, and subcutaneous tissue) and 2% in the extracellular space. This distribution is upheld by the sodium-potassium ATPase and tightly regulated by several mechanisms including those for fluid balance, adrenergic and glycemic control as well as pH buffer systems[1,2,3]. The ratio of intracellular to extracellular potassium is important in determining the cellular membrane potential, and small changes can have profound effects on the function of the cardiovascular and neuromuscular systems. An increase in insulin, catecholamines, aldosterone, and pH shifts potassium from the extracellular to the intracellular space and an increase in blood osmolality and a decrease in pH cause an outward shift. Long term potassium homeostasis is maintained by the kidneys where potassium is freely filtered in the glomeruli and subsequently reabsorbed in the tubular segments proximal to the distal convoluted tubule. In the distal convoluted tubule and collecting ducts potassium is excreted by specialized cells in exchange for sodium. Expression of the responsible ion channels is modified by aldosterone[4].

Extracellular plasma potassium (p-K) measured by blood tests is used for the clinical handling of patients despite it being exceedingly volatile as it is regulated minute-to-minute by various factors mentioned above. Especially considering that, in the acute setting, blood is drawn at a time of extreme stress for the patient, where insulin levels fall and adrenalin levels rise significantly altering p-K while TPK remains unchanged.

We are currently investigating, in a randomised setting, whether it is possible to acutely increase and stabilise potassium at a high high-normal level in order decrease the risk of malignant arrythmias. It is, however, unknown if drugs that alter potassium measured by blood tests only stabilise p-K by means of continues replacement of the potassium in the extracellular space, or if the change in p-K reflects an increased total body potassium. In this substudy, we aim to elucidate this important clinical question.

Whole Body Counter for assessment of total body potassium:

Natural potassium exists as a mixture of three isotopes $^{39}K$, $^{40}K$, and $^{41}K$ with mass percentages of 93.08%, 0.0118% and 6.91% respectively[5]. The radioactive isotope $^{40}K$ emits gamma rays which can be detected by a Whole Body Counter (WBC). The mass percentage distribution of 0.0118% is accurate and reliable and one can therefore extrapolate TBK from these WBC measurements. Potassium measurements *in vivo* by TBK-WBC approach has a long history in research on body composition and nutrition [6].

## Purpose:

1. To investigate if it is possible to increase total body potassium by daily intake of aldosterone antagonists and oral potassium supplements.
2. To investigate if an increase in total body potassium by daily intake of aldosterone antagonists and oral potassium supplements is stable over time.

### Hypothesis:

It is possible to increase total body potassium by daily intake of aldosterone antagonists and oral potassium supplements at clinically relevant doses.

### Methods:

The study is a substudy to the ongoing clinical study "the Potassium trial" at the department of Cardiology in Rigshospitalet and Gentofte Hospitals, and will be conducted in close collaboration with the department of Clinical Physiology at Rigshospitalet. In the Potassium trial, patients are randomized to an intervention that includes daily intake of aldosterone antagonists and oral potassium supplements with the aim to increase p-K to target levels of 4,5-5,0 mmol/l. 14 patients (7 patients randomized for intervention and 7 controls) will be selected to participate in the current substudy. Reference measurements of TBK assessed by WBC (described below) will be performed on two consecutive days at baseline to reduce noise variance. TBK will be measured again after target p-K has been reached in each patient in the intervention group and after 30 days in controls. Finally, TBK will be measured at the first biannual control 6 months after randomisation, in order to examine whether an immediate increase in TBK measured right after intervention is stable over time.

Patients asked to participate in the current substudy will be selected by the Ph.D. student responsible for patient-inclusion to the Potassium trial at Rigshospitalet. Informed consent will be collected and WBC - $^{40}K$ measurement will be scheduled as soon as possible after the initial screening. Patients included will be

randomized to either the intervention or control group after the first $^{40}$K measurement to ensure that baseline measurements are performed before any intervention or group-specific behavioural changes.

## WBC – $^{40}$K measurement:

Before entering the WBC, patients are asked to shower and change into hospital gowns, in order to avoid contamination with radioactive materials in clothing or hair. They then must lie in the WBC in a lead-covered chamber for around 30 minutes as $^{40}$K is measured. $^{40}$K decays directly to $^{40}$Argon with the emission of 1.46 million electron volts (MeV) gamma-rays detected by the WBC. Prior studies have found that the precision of the TBK-WBC approach is 2.3% [7].

## Population:

Patients included in the Potassium trial will be selected for inclusion in the current substudy. The baseline potassium requirements will be set to 4.0 mmol/l or lower, which is stricter than the main study (p-K≤4,3 mmol/l) in order to ensure a satisfactory increase in p-K with intervention. Claustrophobia is added as an exclusion criterion.

**Inclusion criteria:**

Implantable cardioverter defibrillator (ICD) or cardiac resynchronization pacemaker with ICD (CRT-D).

Age >18 years

**Exclusion criteria:**

Estimated glomerular filtration rate (eGFR) <30 ml/h

Pregnancy

Lack of ability to understand and sign informed consent

Claustrophobia

Radiation treatment (e.g. radioiodine therapy) last 6 months.

Nuclear medicine imaging last 3 months.

## Informed consent:

All patients will be informed and sign consent according to the declaration of Helsinki.

## Power calculation:

Preliminary data from the Potassium trial show that the average increase in p-K after intervention is 0,5 mmol/l. To detect an estimated increase in TBK by 5% from an average of 3.850 mmol (55 mEq/kg * 70 kg) with a standard deviation of 125, 14 patients are required (power=0,8, alpha=0,05).

## Ethical and safety concerns:

The current substudy stirs no ethical concerns.

**Risks for the patient:**

Measurements of $^{40}K$ using a WBC is safe and represents no risk for the patients. The only contraindication is claustrophobia and its absence will be ensured at enrollment.

**Benefits for the patient:**

The current substudy has no direct health benefits for the patients included.

## Use of patient data:

This substudy will be conducted in accordance with current Danish legislation on processing of personal data /the General Data Protection Regulation, GDPR in the same way as the main study. The regulations of management of data on individual persons according to the Sundhedsloven og Persondataloven will be followed. Personal data will not be transferred to foreign countries.

## Budget and financing:

Expenses for salary and $^{40}K$-WBC measurements will be applied for through external funds.

Funding is administered through the finance department at The National University Hospital, Rigshospitalet, Copenhagen.

None of the investigators have personal financial interests in the study or personal affiliations with study sponsors.

## Publication of data:

The data will be published in peer-reviewed medical journals after which the data will be de-identified and uploaded to a public database for sharing with other researchers.

Results will be published regardless of whether they are positive, negative or inconclusive.

## Compensation:

The study is covered by Patienterstatningen. Additional insurance is not necessary.

## Study organization:

Initial screening and inclusion will be done at the Department of Cardiology at Rigshospitalet. $^{40}K$ – WBC measurements will be performed at the department of Department of Clinical Physiology, Nuclear Medicine and PET at Rigshospitalet. The study group consists of:

Ulrik Winsløw, MD, Rigshospitalet

Niels Risum, MD, PhD, Rigshospitalet

Christian Jøns, MD. PhD, Rigshospitalet

Henning Bundgaard, Professor, DMSc, Rigshospitalet

Holger Jan Jensen, PhD, Rigshospitalet

Peter S. Oturai, MD, Rigshospitalet

Liselotte Højgaard, Professor, DMSc, Rigshospitalet

## References


1. Bia MJ, DeFronzo RA. Extrarenal potassium homeostasis. *The American journal of physiology*. 1981;240(4):F257-68.

2. ZIERLER KL, RABINOWITZ D. EFFECT OF VERY SMALL CONCENTRATIONS OF INSULIN ON FOREARM METABOLISM. PERSISTENCE OF ITS ACTION ON POTASSIUM AND FREE FATTY ACIDS WITHOUT ITS EFFECT ON GLUCOSE. *The Journal of clinical investigation*. 1964;43:950–62.

3. Silva P, Spokes K. Sympathetic system in potassium homeostasis. *The American journal of physiology*. 1981;241(2):F151-5.

4. BERLINER RW, KENNEDY TJ, HILTON JG. Renal mechanisms for excretion of potassium. *The American journal of physiology*. 1950;162(2):348–67.

5. Lan CY, Weng PS. Body K and 40K in Chinese subjects measured with a whole-body counter. *Health physics*. 1989;57(5):743–6.

6. GB F. Human body composition. *New York: Springer-Verlag;*. 1987.

7. Schneider B, Wang J, Thornton JC, Arbo J, Horlick M, Heymsfield SB PR. Accuracy, Reproducibility, and Normal Total Body Potassium (TBK) Ranges Measured Using the Renovated Whole Body 40K Counter of St. Luke's-Roosevelt Hospital. *International Journal of Body Composition Research*. 2004;(2):51–60.
