# Supplementary material for: Treatment-induced increase in total body potassium in patients at high risk of ventricular arrhythmias; a randomized POTCAST substudy
Source: PLoS One. 2023 Jul 19;18(7):e0288756. doi: 10.1371/journal.pone.0288756 (PMC10355384; doi:10.1371/journal.pone.0288756)
Supplement: S4 File — (PDF) [file pone.0288756.s004.pdf]

Arrhythmia prevention in high risk cardiovascular patients using targeted potassium levels

December 4th 2018, version 5.1

Protocol approval no. H-18044908

Content

1. Title 5

2. Rationale, hypothesis and objective 5

Study Rationale 6

Potassium in cardiovascular disease 6

Hypokalemia and potassium homeostasis 6

Cardioprotective effects of potassium: Arrhythmia prevention 7

Increasing potassium blood levels using aldosterone antagonists and potassium supplementation 8

Whole exome sequencing (WES) and omics 9

Echocardiography – myocardial deformation 10

Hypothesis 10

Objectives 10

3. Methods 11

Study design 11

Randomization 11

Echocardiography 12

Intervention and follow-up 13

Intervention group 13

Control group 14

Device programming 14

ICD tachy-arrhythmia treatment 14

Endpoints 15

Primary endpoint 15

Secondary endpoint 15

Death 16

Heart failure hospital admission 16

Ventricular and supraventricular tachy-arrhythmia therapy 16
Endpoint committee 17
Study time plan 17
Deviation from standard care 17
4. Statistical considerations 17
Population size and accessibility 17
Power calculation 18
Statistical analysis 20
5. Patient population 20
Inclusion criteria 20
Exclusion criteria 20
6. Biological and Genetic material 20
Collection of biological material 20
Genetic material collection and analysis 21
Whole exome sequencing (WES) and omics 21
7. Use of data from patient files 21
Informed consent 22
Data storage and safety 22
Data safety monitoring 22
8. Use of patient data 22
9. Funding and budget 23
10. Reimbursement 24
11. Recruitment of study participants 25
12. Publication of data 25
13. Ethical considerations 26
Risk and benefits of the clinical investigation 26

Anticipated clinical benefits....26

Anticipated clinical risks....26

Interventional medical treatment....27

14. Compensation....27

15. Reference List....27

1. Title

Arrythmia prevention in high risk cardiovascular patients using targeted potassium levels

2. Rationale, hypothesis and objective

Hypokalemia (plasma-potassium (p-K) <3.5 mM) is a frequent encounter in the clinical handling of patients with cardiac diseases. There is solid evidence that potassium-sparing drugs increase survival and ameliorates symptoms in heart failure patients and several post-hoc studies suggest that high-normal levels of p-K decreases the risk of malignant arrhythmias in heart failure patients.

Yet, current guidelines just recommends to keep p-K ≥3.5 mM, a threshold which is below the normal levels in western population (laboratory "normal" reference 3.5-4.9 mM, mean 4.1 mM). No guidelines or recommendations addresses the potential benefits of raising blood levels of potassium to the upper normal levels, e.g. through dietary potassium supplementation, potassium-sparing diuretics etc. Correspondingly, no major trials have addressed this potential.

We suggest a trial randomizing a broad non-selected cohort of patients at high risk of life-threatening cardiac arrhythmia to standard therapy or standard therapy plus a regimen to keep high-normal p-K levels.

Treatment with Implantable Cardioverter-Defibrillator's (ICD's) are offered to patients with a broad range of cardiovascular diseases; ischemic heart disease, heart failure, cardiomyopathies, primary arrhythmia disorders (e.g. long QT syndrome, Brugada syndrome), aborted sudden cardiac death etc., i.e. patients with a high burden of malignant arrhythmia who often receive repeated shock therapies. Thus, ICD treated patients suffer from a considerable unmet medical need and are the target for this study.

Potassium-sparing diuretics and potassium supplements are inexpensive drugs and frequently prescribed in cardiovascular patients to compensate for renal potassium loss related to treatment with thiazide and loop diuretics. Most internists in Denmark have in-depth experience in treatment of the extremes - hypo- and hyperkalemia. This means a positive study outcome can be easily and fast implemented and safely managed by internists and cardiologists.

Study Rationale

Potassium in cardiovascular disease

Low p-K levels are associated with increased morbidity and mortality in patients with heart failure[1] as well as in the general population[2]. High-normal p-K levels have been shown to be associated with an improved outcome compared to normal levels[1], an effect that seems to be present even in mild hyperkalemia[3].

There are several reasons for the improved survival with increasing potassium levels. Most important, potassium plays a crucial role in the formation of the cardiac action potential and with higher levels of p-K, action potential duration shortens and the electrical inhomogeneity decrease causing a marked decrease in the risk of supraventricular and ventricular arrhythmias. Additionally, experimental data suggests a decrease in thrombus formation, atherosclerosis and free radicals with higher levels of potassium and diastolic heart function worsens in hypokalemia[4].

Hypokalemia and potassium homeostasis

Humans evolved on a potassium-rich, sodium-poor diet, and the human body mainly developed mechanisms to retain sodium and excrete potassium corresponding to a daily intake of 50 to 150 mEq (mmol)[5]. Potassium is excreted in the kidneys matching an oral intake from 50 up to 300-400 mmol/day. However, modern Western diets (except the Mediterranean-like diets) may have a potassium content as low as 25 mmol/day. A corresponding increase in sodium intake secondarily increases renal potassium excretion. Hence, modern human diets cause sodium overload and potassium depletion[6] making hypokalemia a common and reversible condition in the Western world.

Virtually all filtered potassium is resorbed in the proximal convoluted tubule and potassium excretion is mainly dependent on the secretion in the distal nephron. Aldosterone and vasopressin stimulate potassium secretion and sodium resorption by upregulating the sodium-potassium-ATPase (a.k.a. the Na,K-pump) and opening luminal sodium and potassium channels. The total body potassium is 3,500 mmol of which 98% is intracellular. Less than 1% of the total body potassium content is locates in the plasma, where potassium is maintained between 3.5 and 5.3 mM by renal excretion and shifts between intracellular and extracellular fluid compartments, by numerous K-channels, transporters and the Na,K-pump in the cell membranes. It has been calculated that the Na,K-pumps in the skeletal muscles have the capacity to clear the potassium from the extracellular phase within seconds. At any given level of potassium content in the body there is a tight

adjustment of levels of p-K, but still the minute to minute levels are influenced by numerous factors during the day including physical activity, insulin, catecholamine and other hormone levels, food intake, hydration level etc.

Due to these regulations it takes a while before a new equilibrium between compartments of the total body potassium content is reached after potassium intake is increased. Due to the effective secretion mechanisms, intake may have to be substantially increased and most likely accompanied by inhibition of aldosterone in order to actively change blood levels within the normal range.

Cardioprotective effects of potassium: Arrhythmia prevention

The transmembraneous gradient of potassium is maintained by the Na,K-pump, which is mainly stimulated by hyperkalemia and aldosterone, but also by exercise, catecholamines and insulin and downregulated by heart failure, hypothyroidism, starvation, diabetes and alcoholism[7]. The gradient between intracellular and extracellular potassium concentrations is one of major determinants of the resting transmembrane potential.

During hypokalemia the resting potential is increased which causes a cellular hyperpolarity and hastens depolarization. This is relatively more pronounced in non-sinus nodal tissue and therefore automaticity and excitability are increased during hypokalemia[8,9]. Hypokalemia causes inhibition of the outward potassium currents in the cardiomyocytes, which prolongs the repolarization phase and leads to dispersion (reflecting electrical inhomogeneity). These changes may present in the ECG as QT prolongation and changes in the morphology of the T wave and as changes in conduction and automaticity resulting in atrial and ventricular ectopy and sustained arrhythmias. Combined, these changes induced by hypokalemia increase the likelihood for development of arrhythmia.

Multiple clinical studies suggest these mechanisms translate to a decrease in spontaneous cardiac arrhythmias with increasing p-K:

In the EMPHASIS-HF study, which included heart failure patients with mild to moderate symptoms, the risk of cardiac endpoints decreased up to a to serum K >5.5 mM (note; serum-K is 0.4-0.5 mM higher than p-K), after which it rose again. Rossignol et al. showed that while sudden death was not significantly reduced in patients treated with eplerenone vs placebo throughout the group, the risk of sudden death was 25% lower in patients who increased s-K >0.11 mM. Furthermore, epleronone was found to decrease the risk of atrial fibrillation[1].

Eplerenone was also used in the EPHESUS study which included sicker heart failure patients. Eplerenone administration resulted in a decrease in mortality and cardiovascular hospitalization

without an increase in the risk for hyperkalemia (p-K >6 mM)[10], and a similar safety profile was shown for spironolactone in the Randomized Aldactone Evalation Study[11].

Even though these studies showed a correlation between increasing p-K and reduced morbidity, the clearer correlation between p-K and mortality was presented by Moss et al., who studied nearly 6.946 patients with heart failure. They identified a clear association between serum K and survival, with the best survival at serum K levels of 5-5.5 mM. Survival was 90% at serum K of 5-5.5 mM and 70% at serum K <3.5 mM as shown in figure 1a.

Bird et al.[12] included patients who underwent stress echocardiography. All patients had blood samples drawn including p-K. Lower values of p-K were associated with an increasing incidence of supraventricular and ventricular arrhythmias during the study as shown in figure 1b

Lumme et al. showed that potassium supplements were given to patients with hypokalemia due to diuretics. As p-K increased, ventricular ectopy ceased[13].

Thus, hypokalemia increases the risk of ventricular and supraventricular arrhythmia and sudden cardiac death and ventricular as well as atrial arrhythmias can be suppressed by raising p-K levels. While this effect seems to be most pronounced in patients with heart failure, it even extends to populations without manifest heart disease[14].

Figure 1a

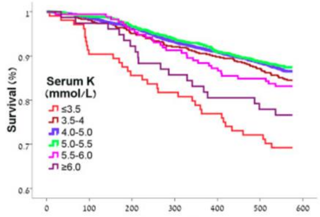

Figure 1b

Potassium, mmol/L	≤3.1	3.2–3.5	3.6–5.2	5.3–5.9	≥6.0
	Dobutamine stress testing (*N* = 13,198)				
n	92	853	11,430	726	97
Transient SVT/AF	5 (5.0%)	41 (4.8%)	487 (4.2%)	16 (2.1%)	1 (1.0%)
> 6 PVC/min	11 (11.0%)	77 (8.9%)	1137 (9.7%)	42 (5.6%)	4 (3.9%)

Figure 1c

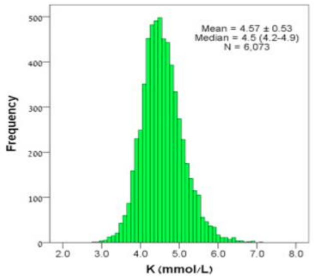

Increasing potassium blood levels using aldosterone antagonists and potassium supplementation

Even though there are no trials actively aiming at maintaining p-K at high-normal levels, useful insight regarding the effect on p-K of various interventions exists from previous trials.

In the EMPHASIS-HF trial, eplerenone was given at a dosage of 25 mg daily to patients with preserved kidney function. P-K increased 0.1-0.2 mM on average during the first couple of months and remained stable for the entire duration of the trial. The risk of hyperkalemia (P-K >5.5 mM)

was 11,2% in the eplerenone-group vs. 7,4% in the placebo group[15] and the risk of p-K >6 mM was minimal.

Similarly, it is striking that also angiotensin converting-enzyme inhibitors, angiotensin II receptor blockers, and beta-blockers all increase plasma K. Thus, a part of the documented clinical outcome of these drugs in patients with cardiovascular diseases might relate to this effect.

Despite the relatively convincing evidence, it has never been tested whether actively keeping the blood level potassium in the upper-normal range in patients with cardiovascular disease is possible and if this decreases the incidence of ventricular and supraventricular arrhythmias.

Whole exome sequencing (WES) and omics

The broad range of cardiovascular diseases necessitating ICD implantation, e.g. ischemic heart disease, heart failure, cardiomyopathies, primary arrhythmia disorders (e.g. long QT syndrome, Brugada syndrome), sustained ventricular tachycardia and aborted sudden cardiac death – in which a specific diagnosis is often not established – have a genetic etiology or a genetic component influencing the development of the disease[16]. Additionally, they have the risk of malignant arrhythmias in common. This may relate not only to the patients disease per se, but also to other common denominators, e.g. genetic pre-dispositions to arrhythmia[17], triggered by the disease.

Thus, several gene variants have been shown to alter the duration of the repolarization phase, and thereby modulate the likelihood of arrhythmia. Most of these variants are not considered disease-causing, but may be important modifiers or even triggers of arrhythmia in patients suffering from other heart diseases. WES is considered useful for identification of variants associated with increased likelihood of arrhythmia, and to identify variants most likely to an additional “hit” for development of arrhythmia in patients with hypokalemia.

In otherwise stable patients with ICD’s, the trigger of a new shock therapy most often remains unidentified. Subclinical infections, inflammatory processes, developing hypertrophy and increasing fibrosis formation[18] possibly related to local tropic factors etc. have been suggested as triggers, but remain to be confirmed. The purpose of genomic analysis in this study is to identify variants associated with

- Unstable potassium levels and tendency to develop hypokalemia/hyperkalemia.
- Vulnerability towards developing arrhythmias during hypokalemic and hyperkalemic episodes

Echocardiography – myocardial deformation

Prognosis in cardiac disease is closely related to systolic function which is commonly measured as left ventricular ejection fraction (LVEF) by echocardiography. Reduced left ventricular ejection fraction (LVEF) of 35% or less is a well-established independent marker of increased risk of arrhythmias and sudden cardiac death in ischemic patient[19]. LVEF has therefore become the main risk stratification tool for primary prevention ICD implantation. However, only 30 % of patients implanted with an ICD receive appropriate therapy and there is a need for further refinement of current selection criteria[20].

New markers of myocardial deformation based on echocardiography have been suggested to be more sensitive. Measures based on 2D speckle tracking analysis such as Global longitudinal strain (GLS) has proven superior to EF as a measure of LV function and as predictor mortality and cardiac events[21]. Similarly, the myocardial dispersion reflecting the inter-segmental variability in contraction duration, has been associated with increased risk of ventricular arrhythmias in a variety of high risk populations such as cardiomyopathies, ischemic heart disease and LQTS[22]. It has been suggested that myocardial dispersion can reflect the electrical heterogeneity of the myocardium which can be seen in diseases such as LQTS or ARVC. If so, the potential electrical stabilization caused by higher potassium levels may also be reflected in the myocardial contractile dispersion.

There is need to explore such mechanisms and to evaluate advanced echocardiographic deformation analysis for the prediction of arrhythmias in larger study populations. The current study represents a unique opportunity to study the importance of potassium levels for myocardial function and how mechanical heterogeneity may be moderated with different levels of potassium.

Hypothesis

Plasma K in the high-normal range reduces the burden of arrhythmias in patients with cardiac diseases.

Objectives

The objectives of this study are:

1) To investigate whether a stable high-normal level of p-K levels are associated with a decrease in malignant arrhythmias and mortality in ICD patients.

2) To investigate to what extent it is possible to increase and maintain plasma K levels to the upper normal range (4.3-4.9 mM).
3) To investigate whether an increase in plasma K is associated with a decreased risk of loss of biventricular pacing due to atrial fibrillation and ventricular ectopy in patients undergoing cardiac resynchronization therapy
4) To investigate the relationship between plasma K levels and incidence of subtypes of arrhythmias

In addition we want to investigate the genetics associated with:

1) Low-normal and high-normal levels of p-K
2) Risk of cardiac arrhythmias at low levels of p-K

Based on echocardiography substudies will be performed to describe the importance of myocardial deformation for cardiac arrhythmias. The objectives are to:

1) Characterize the influence of p-K levels for mechanical heterogeneity
2) Investigate the association between mechanical dispersion and cardiac arrhythmias
3) Prediction of outcome in patients with ICD using conventional and echocardiographic markers

3. Methods

Study design

The study is a prospective, randomized and open-labelled study. The study enrollment, intervention and follow-up cascade is shown in figure 2.

Randomization

Patients will be randomized in a 1:1 ratio to either

a) Usual standard of care
b) Targeted potassium level therapy

Randomization will be controlled by a computer-based algorithm.

Patients undergoing screening with levels of p-K > 4,3 mM are excluded but will be asked to participate in a non-interventional observational study for which endpoints will be obtained from the electronic patient file and the device.

Figure 2:

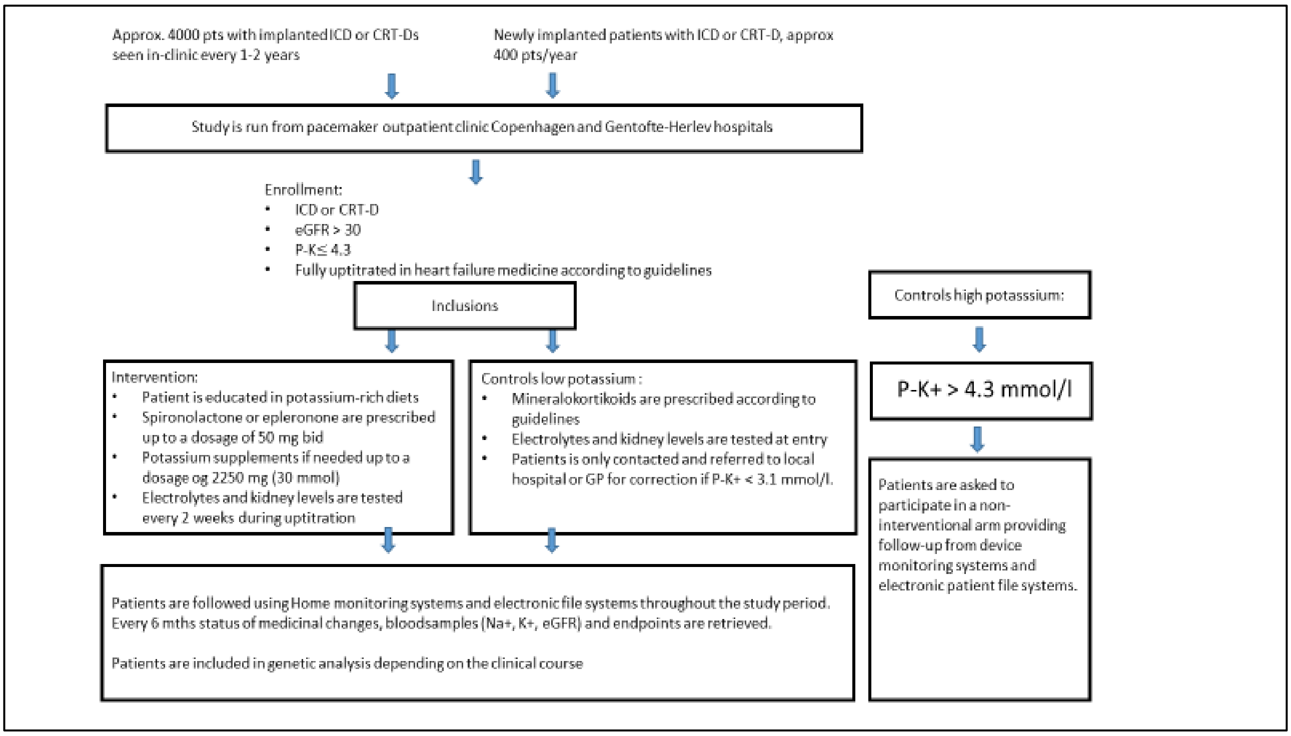

Echocardiography

Patients included in the study will have an echocardiogram performed within 7 days days from inclusion and prior to any intervention. A follow-up echocardiogram will be performed after 6 months. Studies will be analysed for conventional parameters as well as advanced markers of myocardial deformation.

Intervention and follow-up

Intervention group

Blood samples

Plasma levels of K, Na, Mg, Ca, and eGFR and blood pressure and ECG will be measured before and every 2 weeks after dosage adjustment until the p-K target has been reached or maximum study dosages prescribed. At first visit, the blood sample will include genetics and omics.

The patients will be followed bi-annually including measurement of plasma K, Na, Mg, Ca, and eGFR and blood pressure and ECG's.

A study nurse will call the patient every 3 months to ensure compliance and the well-being of the patient

Medical treatment

Written and oral information on potassium rich diets will be provided by the study team. Medical treatment will be up-titrated until a plasma K+ of 4.6-5.0 mM is reached preferably starting with aldosterone antagonist treatment followed by oral potassium supplements if needed (see flow-chart). The final choice, however, will be up to the treating physician.

A p-K up to 5.2 at a single measurement will be accepted during controls without adjustment of medication, but repeated levels of p-K > 5.0 will require medication adjustment.

Patients not already treated with an aldosterone antagonist at a dose of 100 mg/day will be commenced on spironolactone or epleronone according to the choice of the treating physician, with an initial dose of 25 mg od. If plasma K <4,6 mM, spironolactone or epleronone dosages are doubled every 2 week until a maximal dosage of 100 mg under monitoring of blood pressure. If p-K remains <4.6 mM on 100 mg spironolactone or eplerenone, potassium supplements are prescribed commencing at 1500 mg/day up to a maximal dosage of 2250 mg tid.

When maximal dosages of the above mentioned drugs and supplements have been reached or the p-K levels have stabilized between 4.6-5.0 mM the patient will be referred for bi-annual follow-up with their family physician or local hospital, whichever the patient prefers. If eGFR decrease by 30%, if potassium levels become unstable despite regulation the patient should be referred back to the study site. In the case of severe hyperkalemia (p-K > 6.0 mM), the patient should be admitted acutely for treatment.

The treatment cascade is shown in figure 3 below.

Control group

Initiation and adherence to guideline recommended medicine for the disease entity the ICD has been implanted for will be ensured. No other medical treatment interference will be done according to study protocol.

Similar to patients in the intervention group, patients randomized to the control group will be followed bi-annually including measurements of plasma K, Na, Mg, Ca, and eGFR and blood pressure and ECG.

At first visit a blood sample for genetics and omics will be taken.

Figure 3: Treatment cascade.

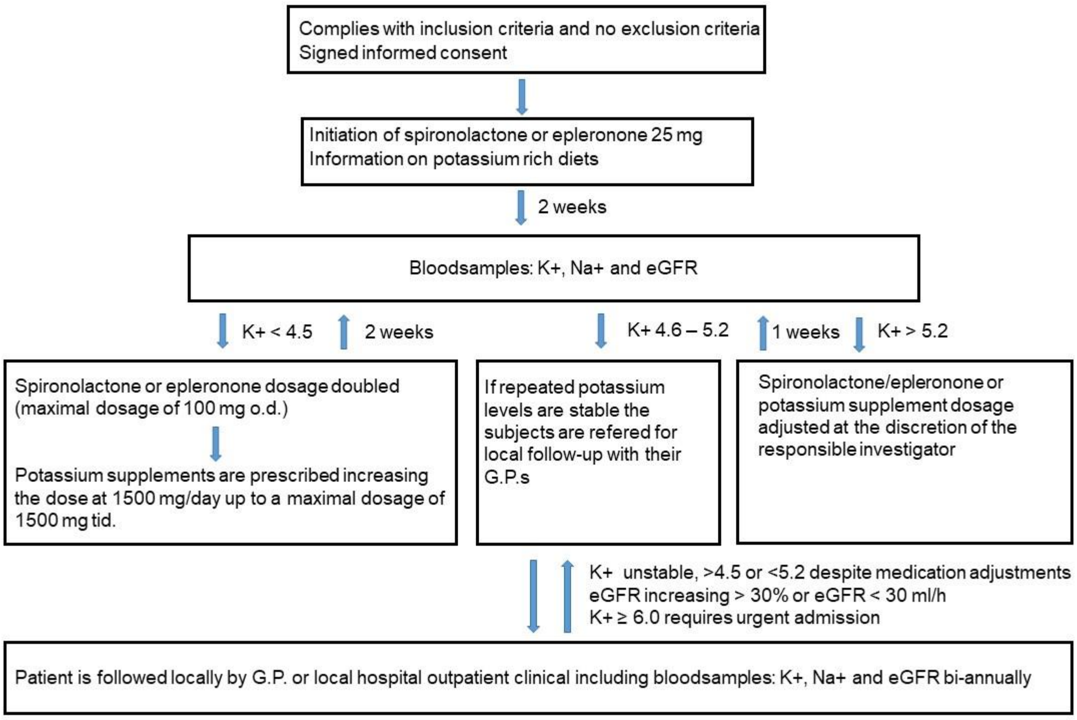

Device programming

ICD tachy-arrhythmia treatment

Home monitoring

All patients with an ICD or CRT-D at the study sites are encouraged to accept home monitoring of the device. In this case, the device will transmit within 24 hours of giving anti-tachycardia or shock therapy. If ventricular tachycardia (VT) or supraventricular tachycardia (SVT) episodes are recorded but not treated, it will be send during a routine 3–monthly transmission, which is standard at both study sites.

Device programming

ICDs from all 5 manufactures are implanted at the study sites and even though modest differences in programming abilities exist between the vendors, devices are programmed relatively identical at the study sites.

A shock, including anti-tachycardia pacing (ATP) during charge is often programmed at heart rates >240 bpm (230-250 bpm). This zone cannot be set to higher levels than 250 bpm and must be set to ON for any other ICD therapy to be delivered.

A fast-VT and a VT zone are available and can be programmed with an individual number of ATP therapies to be delivered before shock therapy is delivered. The fast-VT zone is generally programmed ON with a treatment zone at heart rates 200-240 bpm. Most often 2-4 ATP treatments are delivered before a shock is given. The VT zone is most often used in patients with documented VT and set accordingly at rates starting at 130-180/min with a varying number of ATPs delivered. Shock is only occasionally set ON in this zone.

Hence, ICD units are programmed according to the patient’s condition and recommendations to ICD therapy is not given per protocol

Cardiac resynchronization programming

Cardiac resynchronization therapy (CRT) aims at restoring synchronized contraction in dyssynchronous failing hearts. Successful re-synchronization requires >95% of all heart beats to be paced. This is frequently hampered by atrial fibrillation or frequent ventricular ectopy, and approx. 20% of CRT patients experience periods of low CRT pace for these reasons.

Endpoints

Primary endpoint

A combination of:

- ECG documented ventricular tachycardia > 125 bpm lasting > 30 seconds
- Any appropriate ICD therapy as documented by the ICD
- All cause mortality

Secondary endpoints

- Incidence of supraventricular arrhythmias as documented by ECG or the ICD
- Risk of <92 % CRT-pacing for > 2 weeks (only CRT-D patients)

- Hospitalization for heart failure
- Hospitalization for cardiac arrhythmias
- Hospitalization for electrolyte disturbances or kidney failure
- Appropriate ICD therapy
- Inappropriate ICD therapy

Death

Death events will be collected automatically from the Danish Death registry and the devices will be interrogated post-mortem.

Heart failure hospital admission

Information on hospitalizations will be collected every 6 months according to study protocol. The patient will be asked for hospitalizations.

Ventricular and supraventricular tachy-arrhythmia therapy

Information of ventricular tachyarrhythmia therapy will be obtained from home monitoring systems or interrogation of the ICD devices during in-clinic visits. All ICD therapy is routinely adjudicated as appropriate or in-appropriate by EP physicians at the study sites. All patients receiving an ICD shock are called by the EP personal in the pacemaker outpatient clinic and the circumstances and causes of the shock are investigated and described. Patients receiving successful ATP therapy but not shock are only contacted if is the first time, if a high number of ATP cycles were delivered or if the VT was fast enough to be expected to cause symptoms.

All tachy-arrhythmias causing appropriate ATP or shock or supraventricular tachy-arrhythmias causing inappropriate shock are considered endpoints.

Information of type of therapy delivered, number of therapies, VT/VF duration and cycle length, cause of inappropriate shock (atrial fibrillation, SVT, ICD lead problems) and (if available) circumstances causing the shock (i.e. electrolyte disturbances, will be recorded.

Supraventricular and ventricular arrhythmias not resulting in therapy but documented either because they were of too short duration, triggered mode switch of the device etc will be considered secondary endpoints.

In descriptive studies computer-assisted analyses of digital ECG recordings of QT duration and T wave morphology will be performed to identify markers or patterns associated with risk of future

arrhythmia events. Associations with p-K levels and other parameters (electrolytes, genetic and omics findings) will also be analyzed.

Endpoint committee

An endpoint committee blinded to patient ID will review, adjudicate and classify endpoints. Two reviewers will review an endpoint, if disagreement exists, The endpoint committee will consist of members from the application group, CJ, NR, JHS, HB and TL who are all specialists in cardiac electrophysiology and device interrogation

Study time plan

The study will begin as soon as all necessary approvals have been obtained, with a planned start August[st], 2018.

Patients are recruited over a period of 3 years to allow all patients with ICD implanted to be controlled at the pacemaker clinics. The study is planned to last a total of 4 years.

Additional 1 year is allowed for statistical analysis and publication of the results.

Pt enrollment			Last patients in	Study closing	Publication
	1 yr	2yrs	3yrs	4yrs	5 yrs

|____________|____________|_____________|_____________|______________|

Deviation from standard care

All patients are following the pathway of standard care during the study. As described, patients randomized to the intervention arm will be followed with additional testing until potassium levels are in the targeted interval. Please see the paragraph regarding ethical considerations.

4. Statistical considerations

Population size and accessibility

There are approximately 8.800 patients with an ICD implanted in DK. Approximately 40% of these are followed at Rigshospitalet and at Gentofte Hospital, i.e. approximately 4.000 patients. These are controlled in-clinic every 1-2 years. Hence, a population of approx. 4.000 patients can be screened during the first 2 years. Additionally, 250 new ICDs and 200 new CRT-Ds are implanted at the study sites every year. Of these 60% are primary prophylactic while 40% are secondary

prophylactic. Hence, during an enrollment period of 3 years, a total of 5.350 patients will be available for screening.

Among 7.000 patients with heart failure on relevant medical treatment, median value of p-K was 4.5 mM in patients already on heart failure medication and lower for patients without heart failure. Hence meaning that around 50% of patients can be expected to present with a p-K <4.3 mM (figure 1c).

Severe chronic renal failure is a relative contraindication for implantation of the device because of expected lower benefit and higher risk of infections. In a recent investigation of comorbidities in Danish ICD patients, only 3% had chronic renal failure as a diagnosis during a previous hospitalization. Even though precise number are not available, eGFR <30 ml is conservatively estimated to be found in 5% of the population.

Hence, it is expected that the total population available for screening over 3 years is 5.350 x 0.45 = 2400 patients.

The genetics and omics studies are descriptive and explorative.

Power calculation

Due to the specific constitution of the population, i.e. a pool of patients screened during 2 years with ad additive smaller population of new implants recruited during year 3, we have chosen to use a traditional rather than sequential design of the study.

The risk of the combined endpoint is expected to be approximately average of 16% per year based on our observations on all Danish ICD patients[23] as shown in figure 3 below:

Figure 3: Incidence of appropriate therapy and all-cause mortality amond ICD patients in Denmark implanted with an ICD for primary or secondary prevention.

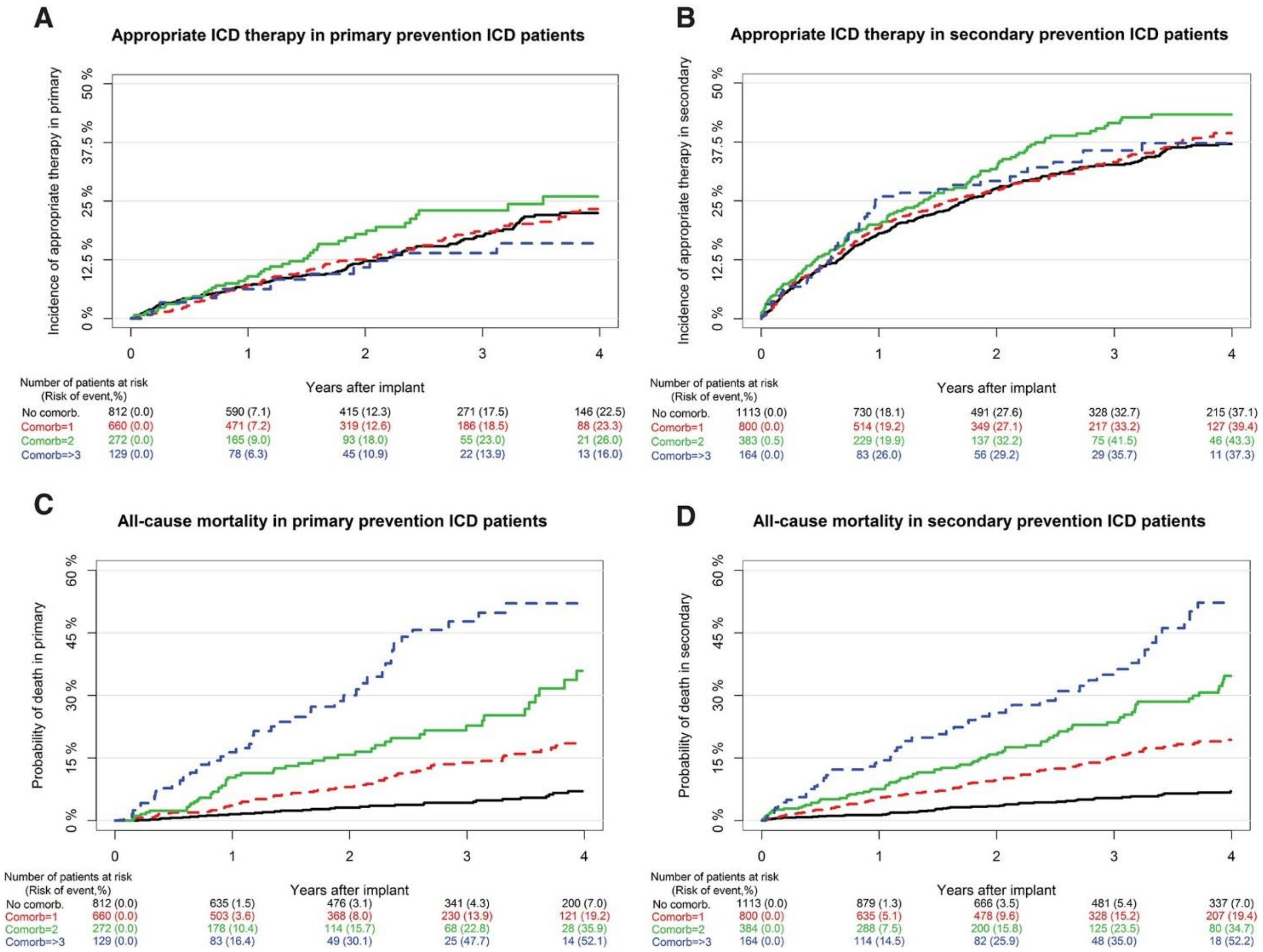

Increasing p-K to 4.4-4.9 mM is believed to reduce therapy rates by approximately 40% based on the observation from Bird et al. Hence, the annual risk of ICD therapy of cardiovascular death is assumed to be 16% in the intervention group and 9.6% in the control group per year.

It is expected that a proportion of patients cannot be brought to a p-K of 4.6. We expect 30% to remain below target values for p-K with a resulting higher risk of the endpoint. This means that 30% remains with unchanged risk and 70% shows an average 40% risk reduction = (30 x 0.16 + 70 x 0.096%), total risk in the treatment group 11.5%, i.e. risk reduction of 1- (11.5 / 16) of 28%, i.e. HR = 0.72

Based on this a total of 291 events are needed. We expect an enrollment phase of 3 years and 1 year additional follow-up to ensure at least 1 year follow-up for all patients, but due to the expected high initial enrollment of the existing pool of ICD patients, we expect an average follow-up of 3 years. Censoring is expected to be low, but is set conservatively at 10%. B

Based on these assumptions, a total of 990 patients should be enrolled. We plan on enrolling a total of 1000 patients.

The power calculation has been overseen by a statistician (Theis Lange).

Statistical analysis

For the purpose of the primary endpoint a time-to-first-event survival analysis will be performed on the primary endpoint. Besides survival statistics, repeated mixed models will be used to understand associations between fluctuations in p-K to drug and potassium supplements therapy as well as the association or p-K to primary and secondary endpoints.

Statistical analysis including for GWAS analysis will be assisted and overseen by a statistician attached to the research group

5. Patient population

Patients will be screened, enrolled and followed from the Outpatient Pacemaker Clinics at Rigshospitalet and Gentofte Hospital. Patients already implanted with ICDs or CRT-Ds are seen in-clinic every 1-2 years and will be screened and enrolled during these follow-ups. Patients admitted for ICD/CRT-D implantation will be screened and enrolled during implant admission or at the 1 month obligatory follow-up.

Inclusion criteria

- Implantable cardioverter defibrillator (ICD) or cardiac resynchronization pacemaker with ICD (CRT-D).
- Age >18 years

Exclusion criteria

- Estimated glomerular filtration rate (eGFR) <30 ml/h
- Pregnancy
- Lack of ability to understand and sign informed consent

6. Biological and Genetic material

Collection of biological material

Acquisition of biological material includes measurements of plasma level K, Na, Mg, Ca, and eGFR before and every 2 weeks after dosage adjustment until the p-K target has been reached or

maximum study dosages prescribed. After analysis these bloodsamples (3 mL x 3) are immediately disposed.

Genetic material collection and analysis

At inclusion a blood sample (3 mL x 3) for genetic analysis and omics will be drawn after a separate specific informed consent for this part of the study.

Biobank

The biological material collected will be stored in a research biobank. Analysis of material from this biobank will take place until 2 years after the last patient has finalized his/her participation in the study, i.e. until December 1st, 2024. Remaining material on this date will be transferred to a biobank on the department for unspecific future research. Remaining material in the biobank in June 30th, 2035 will be destroyed.

Whole exome sequencing (WES) and omics

WES will be applied to identify variants associated with increased likelihood of arrhythmia, and to identify variants most likely to be associated with a positive response to raised p-K levels.

In order to identify triggers of arrhythmic events a subset of patients with repeated un-explained shock therapies will be offered extensive proteomic studies. Stable patients will serve as controls.

7. Use of data from patient files

Data collection and use

Data on clinical characteristics, hospitalizations, genetic findings, treatment outcome and trajectories will be collected. Genetic data will be combined with data from proteomics and transcriptomics studies and protein-protein interaction databases and analyzed using a systems biology approach to identify novel disease genes.

Outcome data such as arhythmia and device therapies will be derived from the device homemonitoring system.

Information used *prior* to inclusion in the study will be confined to:

- Identification of the patient from the patient snapboard, prior to ICD-implantation or prior to routine device-check
- K+ -level and eGFR-level based on routine blood-sample
- Clinical information with regards to functional level and anticongestive medicine.

Information obtained from the potential study participants prior to consent will be passed on to the researchers.

Consent from the study participants will allow investigators, sponsors and sponsor reps as well reseachers with data safety monitoring responsibilities to access any necessary information required to complete the study and for control purposes.

Informed consent

All patients will sign informed consent according to the Helsinki declaration.

Data storage and safety

An electronic database will be set up for the study in REDCAP on the capital region medical research servers (https://redcap.regionh.dk). The database will contain the crude study data for later analysis. This database is approved for containing person-specific data and allows acces on multiple user levels, logging of all access to the data as well as downloading a de-identified version of the database for analysis after study conclusion. The database is hosted and maintained by Center of IT, Medica and Telephony for the Capital Region.

Data safety monitoring

A data safety monitoring board (DSMB) will be constituted before the trial is initiated consisting of 3 independent researches within the field. The purpose of the DSMB is to monitor:

- Interim and cumulative data for evidence of study-related adverse events
- Data quality, completeness, and timeliness
- Performance of the two centers
- Adequacy of compliance with goals for recruitment and retention
- Adherence to the protocol
- Factors that might affect the study outcome or compromise the confidentiality of the trial data (such as protocol violations, unmasking, etc.)
- Factors external to the study such as scientific or therapeutic developments that may impact participant safety or the ethics of the study.

8. Use of patient data

The current study will be conducted in accordance with current Danish legislation on processing of personal data /the General Data Protection Regulation, GDPR.

The Data Protection Agency will be applied for approval, including approval for keeping the genetic data after the project is finalised. The regulations of management of data on individual persons according to the Sundhedsloven og Persondataloven will be followed.

Personal data will not be transferred to foreign countries.

9. Study organization, funding and budget

Study initiators

MD, PhD, Christian Jøns, The Heart Centre B 2142, The National University Hospital, Rigshospitalet, Copenhagen, Blegdamsvej 9, 2100 Copenhagen Ø, Denmark, Phone + 45 3545 9863

Professor, senior consultant, dr.med. Henning Bundgaard, The Heart Centre B 2142, The National University Hospital, Rigshospitalet, Copenhagen, Blegdamsvej 9, 2100 Copenhagen Ø, Denmark, Phone + 45 3545 0512

Professor Søren Brunak, Center for Protein Research, University of Copenhagen, Copenhagen, Blegdamsvej 3, 2200 Copenhagen N, Denmark

MD, PhD, Niels Risum, The Heart Centre B 2142, The National University Hospital, Rigshospitalet, Copenhagen, Blegdamsvej 9, 2100 Copenhagen Ø, Denmark, Phone + 45 3545 9863

Study organization:

The study will be performed at Rigshospitalet and Gentofte/Herlev Hospitals. The study group consists of:

Christian Jøns, MD. PhD, Rigshospitalet

Henning Bundgaard, Professor, DMSc, Rigshospitalet

Jesper Hastrup Svendsen, Professor, DMSc, Rigshospitalet

Niels Risum, MD, PhD, Rigshospitalet

Michael Winther, MD, PhD, Rigshospitalet

Berit Philbert, MD, PhD, Rigshospitalet

Tommi Bo Lindhardt, MD, PhD, Gentofte/Herlev hospitals

Kasper Iversen, MD, DMSc, Gentofte/Herlev hospitals

Juliane Theilade, MD, DMSc, Gentofte/Herlev hospitals

Funding will be applied for through external funds.

Funding is administered through the finance department at The National University Hospital, Rigshospitalet, Copenhagen.

None of the investigators have personal financial interests in the study or personal affiliations with study sponsors.

A specialized nurse technician will be hired specifically for the study at each of the study sites. The study technicians/nurses will screen up to 5-10 potentially recruitable patients per day. They will inform and include patients, take blood samples, take ECG's and blood pressure and manage the bi-annual follow-up. Additionally, they will manage all needed registrations.

Not until the majority of patients are recruited (year 3) a PhD student is employed to plan and execute the data analysis and publication of the results.

Fees for the DSMB are included in the budget.

Salary for a statistical consult is included in the budget.

The endpoint committee consists of members of the application and are not payed.

No medication costs are included as we expect that these (low) costs are covered by the Danish public health care system.

Funding corresponding to 20% of a full clinical time for a clinical cardiologist is included in the budget

10. Reimbursement

There is no reimbursement of expenses for transportation, lack of earnings or drawback allowance for study participants.

11. Recruitment of study participants

A continuous screening for potential study participants will take place during the study period.

A list of patients with a planned visit seven days ahead at the outpatient device clinic at the two centers will be requested from the treating electrophysiologist planned to be in the device clinic at that day. Seven days prior to the planned outpatient visit in the device clinic, the research nurse will send the study information package either via electronic e-post to the patients “e-boks” or, if the patient has requested materiel from the device clinic to be sent via ordinary mail, as physical mail to the patient. The letter will inform the patient about the study and the possibility to receive further information after the normal ICD check-up has been done. The information package will contain 1) recruitment letter, 2) Patient study information and informed consent, 3) the form “Forsøgspersoners rettigheder 2018” and 4) the form “Før du beslutter dig”.

At the device clinic visit the patient will be asked by the pace technician if they have read the study information package and will be interested in additional information.

The oral information will be given in an un-disturbed room by appointed study personal (i.e study nurse, technician or physician who have received training for this purpose). The patient is encouraged to invite an assessor to participate at the meeting. The patient will be offered at least 24 hours to consider his/her participation, i.e. from oral and written information is given and until the patient signs the consent form. If the patient consents to participate in the study, the bloodwork necessary for randomization will be done following the oral information. If the necessary bloodwork has been obtained within 7 days during another un-related clinical visit, these test results will be used and the bloodwork will not be repeated.

For each person his/her mental and physical integrity and rights of privacy will be respected.

12. Publication of data

The data will be published in peer-reviewed medical journals after which the data will be de-identified and uploaded to a public database for sharing with other researchers.

Results will be published regardless of whether they are positive, negative or inconclusive. The study will be announced at www.clinicaltrials.gov

13. Ethical considerations

Risk and benefits of the clinical investigation

Anticipated clinical benefits

Besides from the assumed positive effects of high-normal K+ levels in the interventional group, patients in both groups followed in the study will be monitored more often compared to what is usual practice, and a cardiac specialist will monitor optimal medical treatment at the beginning and throughout the trial. Many of the patients enrolled in this trial will not have been seen by a cardiologist for several years.

Anticipated clinical risks

The risk of hyperkalemia is likely to be increased in the intervention group. In a recent meta analysis on the risk of hopitalizations for hyperkalemia associated with mineralocorticoids in heart failure, the risk of hyperkalemia was doubled[24] (epleronone 5.0 vs 2.6%, spironolactone 17.5 vs 7.5%). On the other hand, the risk of hospitalization for hypokalemia is decreased with 50%. In general, the risk associated with hypokalemia was associated with a higher risk of death (see figure 2a and 2b, adapted from Núñez et al[25]). Hence, we consider the lower risk of hypokalemia along with a greater risk of hyperkalemia as being an advantage for the patient.

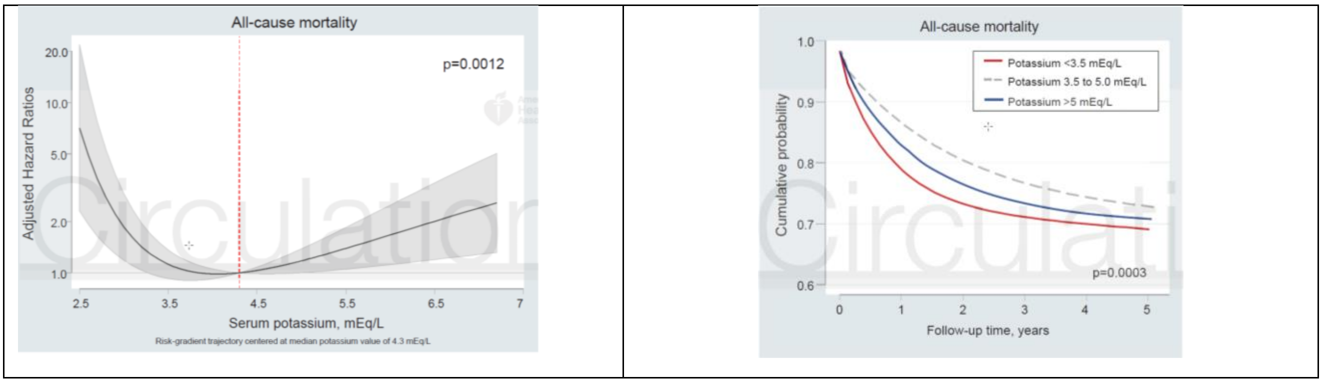

Figure 2a and 2b: Risk of death associated with having a blood sample collected with hypokalemia or hyperkalemia.

In a study investigation on reasons and management of hyperkalemia, mineralocorticoid treatment was the most frequent cause[26]. Among 600 hospitalizations, by far most of them were mild hyperkalemia and treated with cessation of medication and fluid therapy whereas a little less than 8% required hemodialysis. There were no deaths.

In both groups additional discomfort from bloodsampling can be expected.

Interventional medical treatment

Potassium supplements are available over-the-counter in Denmark. Treatment with potassium supplements and diet recommendations are considered safe.

The aldosterone antagonists spironolactone and epleronone are prescribed with indication of heart failure with symptoms corresponding to NYHA II- IV, liver failure and for treatment of hypertension. The major side effects are related to hyperkalemia. This risk is low in patients with normal to slightly reduced kidney function. P-K levels will be monitored at intervals short enough to reduce the risk of hyperkalemia episodes to a minimum. Hence, the risk of developing severe hyperkalemia induced arrhythmia is therefore low. If arrhythmias develop, the patients have an ICD implanted and the investigators will be notified immediately since all patients will be using home monitoring.

14. Compensation

This study is covered by Patienterstatningen. Additional insurance is not necessary.

15. References

1. Hoss S, Elizur Y, Luria D, Keren A, Lotan C, Gotsman I. Serum Potassium Levels and Outcome in Patients With Chronic Heart Failure. *Am J Cardiol*. 2016;118:1868–1874.

2. Krogager ML, Torp-Pedersen C, Mortensen RN, Køber L, Gislason G, Søgaard P, Aasbjerg K. Short-term mortality risk of serum potassium levels in hypertension: a retrospective analysis of nationwide registry data. *Eur Heart J*. 2017;38:104–112.

3. Ahmed MI, Ekundayo OJ, Mujib M, Campbell RC, Sanders PW, Pitt B, Perry GJ, Bakris G, Aban I, Love TE, Aronow WS, Ahmed A. Mild hyperkalemia and outcomes in chronic heart failure: a propensity matched study. *Int J Cardiol*. 2010;144:383–388.

4. Macdonald JE, Struthers AD. What is the optimal serum potassium level in cardiovascular patients? *J Am Coll Cardiol*. 2004;43:155–161.

5. Dahl LK. Salt intake and salt need. *N Engl J Med*. 1958;258:1152-1157 contd.

6. Meneely GR, Battarbee HD. High sodium-low potassium environment and hypertension. *Am J Cardiol*. 1976;38:768–785.

7. Clausen T. Na+-K+ pump regulation and skeletal muscle contractility. *Physiol Rev*. 2003;83:1269–1324.

8. Fisch C, Knoebel SB, Feigenbaum H, Greenspan K. Potassium and the monophasic action potential, electrocardiogram, conduction and arrhythmias. *Prog Cardiovasc Dis*. 1966;8:387–418.

9. Gettes L, Surawicz B. Effects of low and high concentrations of potassium on the simultaneously recorded Purkinje and ventricular action potentials of the perfused pig moderator band. *Circ Res*. 1968;23:717–729.

10. Pitt B, Bakris G, Ruilope LM, DiCarlo L, Mukherjee R, EPHESUS Investigators. Serum potassium and clinical outcomes in the Eplerenone Post-Acute Myocardial Infarction Heart Failure Efficacy and Survival Study (EPHESUS). *Circulation*. 2008;118:1643–1650.

11. Pitt B, Zannad F, Remme WJ, Cody R, Castaigne A, Perez A, Palensky J, Wittes J. The effect of spironolactone on morbidity and mortality in patients with severe heart failure. Randomized Aldactone Evaluation Study Investigators. *N Engl J Med*. 1999;341:709–717.

12. Bird JG, McCully RB, Pellikka PA, Kane GC. Dobutamine Stress Echocardiography: Impact of Abnormal Blood Potassium Levels on Cardiac Arrhythmias. *J Am Soc Echocardiogr*. 2017;30:595–601.

13. Lumme JA, Jounela AJ. The effect of potassium and potassium plus magnesium supplementation on ventricular extrasystoles in mild hypertensives treated with hydrochlorothiazide. *Int J Cardiol*. 1989;25:93–97.

14. Mattsson N, Sadjadieh G, Kumarathurai P, Nielsen OW, Køber L, Sajadieh A. Ambulatory cardiac arrhythmias in relation to mild hypokalaemia and prognosis in community dwelling middle-aged and elderly subjects. *Europace*. 2016;18:585–591.

15. Zannad F, McMurray JJV, Krum H, van Veldhuisen DJ, Swedberg K, Shi H, Vincent J, Pocock SJ, Pitt B, EMPHASIS-HF Study Group. Eplerenone in patients with systolic heart failure and mild symptoms. *N Engl J Med*. 2011;364:11–21.

16. Cirino AL, Harris S, Lakdawala NK, Michels M, Olivotto I, Day SM, Abrams DJ, Charron P, Caleshu C, Semsarian C, Ingles J, Rakowski H, Judge DP, Ho CY. Role of Genetic Testing in Inherited Cardiovascular Disease: A Review. *JAMA Cardiol*. 2017;2:1153–1160.

17. Christophersen IE, Rienstra M, Roselli C, Yin X, Geelhoed B, Barnard J, Lin H, Arking DE, Smith AV, Albert CM, Chaffin M, Tucker NR, Li M, Klarin D, Bihlmeyer NA, Low S-K, Weeke PE, Müller-Nurasyid M, Smith JG, Brody JA, Niemeijer MN, Dörr M, Trompet S, Huffman J, Gustafsson S, Schurmann C, Kleber ME, Lyytikäinen L-P, Seppälä I, Malik R, Horimoto ARVR, Perez M, Sinisalo J, Aeschbacher S, Thériault S, Yao J, Radmanesh F, Weiss S, Teumer A, Choi SH, Weng L-C, Clauss S, Deo R, Rader DJ, Shah SH, Sun A, Hopewell JC, Debette S, Chauhan G, Yang Q, Worrall BB, Paré G, Kamatani Y, Hagemeijer YP, Verweij N, Siland JE, Kubo M, Smith JD, Van Wagoner DR, Bis JC, Perz S, Psaty BM, Ridker PM, Magnani JW, Harris TB, Launer LJ, Shoemaker MB, Padmanabhan S, Haessler J, Bartz TM, Waldenberger M, Lichtner P, Arendt M, Krieger JE, Kähönen M, Risch L, Mansur

AJ, Peters A, Smith BH, Lind L, Scott SA, Lu Y, Bottinger EB, Hernesniemi J, Lindgren CM, Wong JA, Huang J, Eskola M, Morris AP, Ford I, Reiner AP, Delgado G, Chen LY, Chen Y-DI, Sandhu RK, Li M, Boerwinkle E, Eisele L, et al. Large-scale analyses of common and rare variants identify 12 new loci associated with atrial fibrillation. *Nat Genet*. 2017;49:946–952.

18. Nguyen M-N, Kiriazis H, Gao X-M, Du X-J. Cardiac Fibrosis and Arrhythmogenesis. *Compr Physiol*. 2017;7:1009–1049.

19. Epstein AE, Dimarco JP, Ellenbogen KA, Estes NAM, Freedman RA, Gettes LS, Gillinov AM, Gregoratos G, Hammill SC, Hayes DL, Hlatky MA, Newby LK, Page RL, Schoenfeld MH, Silka MJ, Stevenson LW, Sweeney MO, American College of Cardiology/American Heart Association Task Force on Practice, American Association for Thoracic Surgery, Society of Thoracic Surgeons. ACC/AHA/HRS 2008 guidelines for Device-Based Therapy of Cardiac Rhythm Abnormalities: executive summary. *Heart Rhythm*. 2008;5:934–955.

20. Buxton AE, Lee KL, Hafley GE, Pires LA, Fisher JD, Gold MR, Josephson ME, Lehmann MH, Prystowsky EN, MUSTT Investigators. Limitations of ejection fraction for prediction of sudden death risk in patients with coronary artery disease: lessons from the MUSTT study. *J Am Coll Cardiol*. 2007;50:1150–1157.

21. Stanton T, Leano R, Marwick TH. Prediction of all-cause mortality from global longitudinal speckle strain: comparison with ejection fraction and wall motion scoring. *Circ Cardiovasc Imaging*. 2009;2:356–364.

22. Haugaa KH, Smedsrud MK, Steen T, Kongsgaard E, Loennechen JP, Skjaerpe T, Voigt J-U, Willems R, Smith G, Smiseth OA, Amlie JP, Edvardsen T. Mechanical dispersion assessed by myocardial strain in patients after myocardial infarction for risk prediction of ventricular arrhythmia. *JACC Cardiovasc Imaging*. 2010;3:247–256.

23. Ruwald AC, Vinther M, Gislason GH, Johansen JB, Nielsen JC, Petersen HH, Riahi S, Jons C. The impact of co-morbidity burden on appropriate implantable cardioverter defibrillator therapy and all-cause mortality: insight from Danish nationwide clinical registers. *Eur J Heart Fail*. 2017;19:377–386.

24. Vukadinović D, Lavall D, Vukadinović AN, Pitt B, Wagenpfeil S, Böhm M. True rate of mineralocorticoid receptor antagonists-related hyperkalemia in placebo-controlled trials: A meta-analysis. *Am Heart J*. 2017;188:99–108.

25. Núñez J, Bayés-Genís A, Zannad F, Rossignol P, Núñez E, Bodí V, Miñana G, Santas E, Chorro FJ, Mollar A, Carratalá A, Navarro J, Górriz JL, Lupón J, Husser O, Metra M, Sanchis J. Long-Term Potassium Monitoring and Dynamics in Heart Failure and Risk of Mortality. *Circulation*. 2018;137:1320–1330.

26. Eliacik E, Yildirim T, Sahin U, Kizilarslanoglu C, Tapan U, Aybal-Kutlugun A, Hascelik G, Arici M. Potassium abnormalities in current clinical practice: frequency, causes, severity and management. *Med Princ Pract*. 2015;24:271–275.
