# Supplementary material for: Treatment-induced increase in total body potassium in patients at high risk of ventricular arrhythmias; a randomized POTCAST substudy
Source: PLoS One. 2023 Jul 19;18(7):e0288756. doi: 10.1371/journal.pone.0288756 (PMC10355384; doi:10.1371/journal.pone.0288756)
Supplement: Attachment — Submitted filename: Response to Reviewers.docx [file pone.0288756.s005.docx]

Reviewer #1: The manuscript addresses a potentially interesting topic. The study is single-center and this may limit its usefulness. Similarly, the methods are rather basic and not fully discussed. Some comments follow.

1. Please, upload the code used to get the results. This ensures the reproducibility of the results and allows the reviewer to check for the correctness of the results.

Response: Certainly. The code for main results is presented below.

Using the Statistics software R version 4.1.0 and the open-source package lme4:

#loading package for mixed linear regression analysis
library(lme4)	# "golden standard" for mixed-effects modelling in R

#Creating a dataframe with long format data required for mixed regression analysis.
glm_tbk<-c(tbk_baseline,tbk_6weeks, tbk_6months)
glm_pk<-c(pk_baseline, pk_6weeks,pk_6months)
glm_fu<-gl(3,14)
glm_fu<-as.numeric(glm_fu)
glm_pt_nr<-c(1:14,1:14,1:14)
glm_group<-c(group, group, group)
glm_group<-as.factor(glm_group)
glm_koen<-c(koen, koen, koen)
glm.data<-data.frame(glm_tbk, glm_pk, glm_fu, glm_pt_nr, glm_group)

#changing the unit of glm_tbk from g/kg to mg/kg
glm.data$glm_tbk<-glm.data$glm_tbk*1000

#changing class of glm_fu and glm_group to factor
glm.data$glm_fu<-as.factor(glm.data$glm_fu)
glm.data$glm_group<-as.factor(glm.data$glm_group)

#linear mixed regressio nmodel with patient ID (glm_pt_nr) as random effect.
lmer<-lmer(glm_tbk~glm_fu*glm_group+(1|glm_pt_nr), data=glm.data3, REML=F)

#visual inspection of residual normality
qqnorm(resid(lmer))
qqline(resid(lmer))

#formal statistical test for residual normality
shapiro.test(resid(lmer))

#extracting model results
summary(lmer)
confint(lmer)
coef(lmer)

#linear mixed regression model of p-K with patient ID (glm_pt_nr) as random effect.
lmer2<-lmer(glm_pk~glm_fu*glm_group+glm_group+(1|glm_pt_nr), data=glm.data3, REML=F)

#visual inspection of residual normality
qqnorm(resid(lmer2))
qqline(resid(lmer2))

#formal statistical test for residual normality
shapiro.test(resid(lmer2))

#extracting model results
summary(lmer2)
confint(lmer2)
coef(lmer2)

2. The sample size is rather small. This may strongly affect statistical inference. Even though basic tests are employed, no matter which parametric approach is considered, the assumptions underlying the statistical methods must be carefully checked and discussed.
3. I appreciate the use of mixed effects modelling as the data have a clear longitudinal structure. Nevertheless, the results are not well discussed. Firstly, it is rather unclear what the outcome is. The paper discusses mean differences at multiple times, and the regression modelling is swept under the carpet. Again, model's assumptions must be checked, investigated and discussed. A residual analysis must be provided and results must be shown to support your statements. Moreover, I am wondering how the Gaussian assumption for the random effects is tenable for such a small sample. Overall, it is rather unclear if confounders are considered or not, and the empirical specification of the linear predictor is not discussed.

Response: Thank you for these two important comments on sample size and statistical modelling. We agree that checking the assumptions behind any statistical test is of vital importance. The underlying test for the main results in the present paper is the linear mixed random effects regression model and the assumptions was tested during data analysis. The code is shown in comment 1. We have included a residual analysis in the manuscript:

Page 7, line 167: The assumption of normal distribution of model residuals was tested by Shapiro-Wilk test. For changes in p-K the W statistic was 0.97, P=0.39 and for TBK the W statistic was 0.96, P=0.11.

The outcome was predefined as between-group difference in changes in p-K and TBK from baseline to six weeks and from baseline to six months. This has been made clearer on page 6 line 134: **Main outcome measures:** Between-group difference in changes in TBK and p-K from baseline to six weeks, and from baseline to six months.

As for confounding factors and generalizability, the study is a randomized trial and such trial of its kind. However, we do agree that the relatively low sample size, the between-study heterogeneity, may affect the generalizability of the results. This has now been addressed in the limitations section on page 14 line 276:

Changes made to the manuscript:

Due to the low sample size and heterogeneous study population, larger studies with a number of subgroups are needed to confirm the generalizability of the findings.

Reviewer #2: This is an interesting study considering that serum potassium level is significantly associated with the cardiovascular event risk. Another important point suggested from the study is that p-K level and TBK are rather differently regulated and do not behave parallelly in the living body homeostasis.
However, the reviewer has concerns as below.

#1. Only value differences were plotted in Figure 2. The measurement values should definitely be separately presented in each group with statistical standard deviations.

Response: Thank you for this comment. We chose to keep the study simple by only presenting differences in outcome between the two groups in the main paper. We realize that some readers may be interested to know the changes in each group separately along with standard deviations and this data has been added to a table in the supplementary appendix (Supplementary table 1). We have added a sentence to the manuscript in order to refer readers to the table on page 11 line 207.

Changes made to the manuscript:

The mean TBK and p-K in each study group at baseline, six weeks, and six months are shown in S1 Table.

#2.The authors’ idea about the sample size calculation is questionable. The reviewer agree with the idea that the study was powered to detect an increase in TBK of 5%. However, as shown in Supplemental Figure 2, the mean difference between measurements in the same sample was 0.024 g/kg body weight, which may up-to 1.6% of the absolute value. Considering the measurement error, the reviewer can not believe that the sample size calculation is sufficient to interpret the results. The sample size calculation should definitely be revised. I guess more number of samples should be included to lead a conclusion.

Response: Thank you for this comment. The sample size calculation was performed prior to the initiation of the study and thus, naturally holds estimations of the study outcome. As no prior trial has been made on the same study population some assumptions had to be made. Thus, we assumed that …… and standard deviations for the sample size calculation were achieved from ….

We all prefer large studies – but we also have to acknowledge the ethics - that it is considered un-ethical to expose more patients to trial examinations than what has been shown to be necessary to reach a conclusion in the specific study.

The observed changes came quite close to the estimated changes. TBK increased slightly more than estimated (0.083 g/kg) while the standard deviation of the paired differences was 0.05. A future study with a sample size calculation based on these numbers would need 6 patients in each group.
