# Supplementary material for: Treatment-induced increase in total body potassium in patients at high risk of ventricular arrhythmias; a randomized POTCAST substudy
Source: PLoS One. 2023 Jul 19;18(7):e0288756. doi: 10.1371/journal.pone.0288756 (PMC10355384; doi:10.1371/journal.pone.0288756)
Supplement: Attachment — Submitted filename: Response to Reviewers.docx [file pone.0288756.s006.docx]

**Response to editor:**

We are very pleased that you found our responses to the reviewers acceptable.

As for the editor’s comment on adding a sentence about sample size to the ethics section, we have modified the manuscript with the following on page 7 line 160:

“The limited size of the study was solely based on the sample size calculation as it is, due to ethical considerations, recommended to avoid exposing more patients to trial examinations than what has been shown to be necessary to reach a conclusion.“
